# Effectiveness of tetracycline fibers as an adjunct to scaling and root planing in chronic periodontitis

**DOI:** 10.6026/973206300221137

**Published:** 2026-02-28

**Authors:** Mohammad Arifkhan, Neetu Rani, Shailja Singh, Mohd Ayub Siddiqui, Sachin Kumar, Mohsin Khan, Ganiga Channaiah Shivakumar

**Affiliations:** 1 Department of Periodontology, Career Post Graduate Institute of Dental Science, Lucknow, Uttar Pradesh, India; 2 Department of Periodontology, Faculty of Dental Sciences, KGMU, Lucknow, Uttar Pradesh, India; 3 Department of Conservative Dentistry and Endodontics, KGMU, Lucknow, Uttar Pradesh, India; 4 Department of Oral and Maxillofacial Surgery, Career Post Graduate Institute of Dental Sciences and Hospital, Lucknow, Uttar Pradesh, India; 5 Department of Oral and Maxillofacial Surgery, School of Dental Sciences, Sharda University, Greater Noida, Uttar Pradesh, India; 6 Department of Oral Medicine & Radiology, People's College of Dental Sciences and Research Centre, People's University, Bhopal, India

**Keywords:** Tetracycline, local drug delivery system, chronic periodontitis, scaling and root planing, clinical study

## Abstract

Chronic periodontitis remains highly prevalent and scaling and root planing alone may be insufficient to achieve optimal clinical improvement at moderate periodontal pocket depths. Therefore, it is of interest to clinical efficacy of scaling and root planing alone with scaling and root planing combined with tetracycline-impregnated collagen fibers in patients with chronic periodontitis. Thirty patients (60 sites) with probing depths of 4-7 mm were treated using a split-mouth design and plaque index, gingival index, probing depth and clinical attachment level were recorded at baseline and follow-up. Both groups showed significant improvement in clinical parameters, with the test group demonstrating greater probing depth reduction and clinical attachment gain at 90 days compared to controls. Adjunctive use of tetracycline-impregnated collagen fibers enhanced the clinical outcomes of scaling and root planing in the short-term management of chronic periodontitis.

## Background:

Periodontitis is an inflammatory disease of the supporting tissues of the teeth caused by specific microorganisms or groups of specific microorganisms, resulting in progressive destruction of the periodontal ligament and alveolar bone with pocket formation, recession, or both [1]. Periodontitis is one of the most prevalent chronic diseases in the world, with the primary etiological agent being pathogenic bacteria that reside in the subgingival area [2]. American Academy of Periodontology (AAP) held an international workshop in 1999 to introduce a classification system based on current clinical and scientific data. The resulting classification of the different forms of periodontitis was simplified to describe three general clinical manifestations of periodontitis. They are chronic periodontitis, aggressive periodontitis and periodontitis as a manifestation of systemic diseases. Chronic periodontitis is most prevalent in adults but can be observed in children. Clinical features are gingival inflammation, pocket formation, loss of periodontal attachment, loss of alveolar bone and occasionally suppuration [1]. The main goal of periodontal therapy is not only to stop periodontal destruction but also to prevent the recurrence of disease and regeneration of lost tissues [3]. Mechanical debridement alone may fail to eliminate the putative pathogens from the pockets completely because of the location of these organisms within gingival tissue or in deeper areas inaccessible to periodontal instrumentations and thus results in recurrence of disease. Therefore, the selective removal or inhibition of pathogenic microbes with systemic or topical antimicrobial agents in combination with scaling and root planing (SRP) is often considered as an effective approach at specific disease active sites. Various local delivery methods for administering antimicrobial agents directly into the periodontal pocket have been tested [4]. These methods further minimize the side effects of systemically administered drugs and also maintain a high level of antimicrobial agents within the gingival crevicular fluid over an extended period. Local drug delivery avoids most adverse reactions and disadvantages with little or no systemic effects. The local concentration achieved may be much higher than that is possible through the systemic route [5]. Among the antimicrobials used in periodontal therapy, much attention has been focused on tetracycline. Tetracycline offers a broad- spectrum antimicrobial activity and may be a useful adjunct to periodontal therapy [6]. Therefore, it is of interest to evaluate and compare the changes in clinical index and clinical parameter of SRP treated sites alone with sites treated by SRP along with the adjunctive tetracycline fibers.

## Materials and Methods:

## Selection criteria:

## Study design:

The study populations comprised of individuals who were having chronic periodontitis, between the age group of 35-60 comprised of both sexes and the sites must have pockets measuring 4-7 mm clinically and had not received any surgical or non-surgical periodontal therapy were enrolled in the study. A total number of 60 sites from 30 patients with periodontal pocket depth measuring 4-7 mm on contra lateral sides were selected for a split mouth trial.

The sites selected were divided into;

[1] Control Site-30 sites in number. Treated with scaling and root planing.

[2] Test Site -30 sites in number. Treated with scaling and root planing followed by placement of Periodontal Plus AB® (Tetracycline- fiber).

## Inclusion criteria:

[1] Patients with two non-adjacent sites located in separate quadrants that required periodontal treatment.

[2] Subjects between the age group of 35 to 60 years of age.

[3] No history of any surgical or non-surgical periodontal therapy.

[4] The sites must have pockets measuring 4-7 mm clinically and demonstrated radiographic evidence of moderate alveolar bone loss.

## Exclusion criteria:

[1] Pregnant & lactating women.

[2] Patients with known hypersensitivity to tetracycline.

[3] Patients having teeth with furcation involvement.

[4] Patients with a history of smoking and drug or alcohol abuse.

[5] Patients who have received antibiotics within the preceding 3 months of the study.

## Clinical parameters used for assessment:

[1] Plaque score using Plaque Index (PI; Silness and Loe1964)78

[2] Gingivitis using Gingival Index (GI; Loe and Silness1963)79

[3] Periodontal Probing depth: Measured by UNC-15 probe. 80

## Probing pocket depth:

Probing depth were measured at mesio - buccal, buccal, disto - buccal, mesio - lingual, lingual, disto - lingual surfaces of the tooth using a UNC 15 probe from the crest of marginal gingiva to the base of the pocket and recorded to the nearest millimeter.

## Method of statistical analysis:

Statistical Methods: The descriptive data is presented as Mean + Standard deviation and percentages. Frequency distribution charts and tables are used to know the correlation of the means of the values obtained from the parameters in different groups.

MEAN: It is the sum of all observed results from the sample divided by the total number of events. n = sample size

## Statistical software:

All the Statistical Analysis were carried out using SPSS version 23.0 were used for the analysis of the data and MS Excel & word 2016 have been used to generate graphs, tables etc.

## Results:

A total of 30 patients i.e., 60 sites from the patients were selected and grouped into two categories: control and test. The control group i.e. GROUP 2 (30 sites) was treated with SRP without using tetracycline-impregnated collagen fibers (Control Site). The test group i.e. GROUP 1 (30 sites) was treated by SRP plus tetracycline-impregnated fibers (Test Site). All the patients were subjected to SRP at the baseline measurement. Prior to SRP each selected site was subjected to assessment of the following parameters; (1) Plaque Index; (2) Gingival Bleeding Index; (3) Clinical Pocket Depth. Thorough SRP was done at both the sites using ultra sonic scalers. In one of the contra lateral sites, commercially available tetracycline impregnated collagen fibers were administered after SRP in test group. For sites also receiving fiber therapy, fibers containing tetracycline were placed in the periodontal pocket surrounding the tooth. Fibers were placed until the pocket was entirely filled to the gingival margin. Patients were instructed to avoid brushing the fiber-treated teeth and eating crusty foods until fibers and adhesive were removed. At approximately 4 days following placement, the patients were checked for fiber retention and any adverse reaction. Verbal instructions were given to avoid manipulating the study teeth. At each visit (Baseline, 30th, 60th and 90th day), the clinical parameters were assessed. A single trained and calibrated examiner who was blinded to treatment method obtained all readings. Clinical parameters were assessed sequentially. The assessment of plaque was done on the basis of thickness of plaque at the gingival margin area of the sites using plaque index (Sillness and Loe 1964). Gingival index (Loe and Sillness, 1963) was recorded for each site. Pocket depth was measured with the help of UNC 15 probe (Hu -Friedy) and the distance between the base of the pocket and gingival margin was measured. The probe was inserted parallel to the long axis of the tooth and walked circumferentially around each tooth to detect the areas of deepest penetration. As the resistance to further penetration was noticed, readings were recorded to the nearest millimeter. A total of 30 patients enrolled at the baseline completed the evaluation. A total number of 60 sites from 30 patients with periodontal pockets measuring (4-7 mm) in contra lateral quadrants were selected. The selected sites were divided into the Control Group (GROUP 2) treated by SRP alone and the Test Group (GROUP 1) which received SRP and treatment with commercially available tetracycline-impregnated collagen fibers (Table 1). The comparison of mean age between female and male patients showed no statistically significant difference (p = 0.618) using the Independent t-test. The mean age among females was 47.75 ±16.29 years, while among males it was 45.33 ±9.62 years (Table 2). At 30th day, the mean plaque scores of test groups were 0.56 ±0.22 and control group were 0.83 ±0.22. The comparison was done using Wilcoxon paired t test, the difference reached the level of significance. Significantly lower plaque scores were seen in test group as compared to control group at 60th day, the mean plaque scores of test group were 0.94 ±0.34 and control group were 1.13 ± 0.30. The comparison was done using Wilcoxon paired t test, the difference reached the level of significance. Significantly lower plaque scores were seen in test group as compared to control group (Table 3).

At 90th day, the mean plaque scores of test group were 0.86 ±0.35 and control group were 1.04 ±0.34. The comparison was done using Wilcoxon paired t test, the difference reached the level of significance. Significantly lower plaque scores were seen in test group as compared to control group at baseline, the mean gingival scores of test group were 1.44 ±0.23 and control group were 1.55 ±0.21 the comparison was done using Wilcoxon paired t test, the difference failed to reach the level of significance. At 30th day, the mean gingival scores of test group were 1.20 ±0.17 and control group were 1.43 ±0.22. The comparison was done using Wilcoxon paired t test, the difference reached the level of significance. Significantly lower mean gingival scores were seen in test group as compared to control group (Table 4). At 60th day, the mean gingival scores of test groups were 0.91 ±0.27 and control group were 1.25 ±0.23. The comparison was done using Wilcoxon paired t test, the difference reached the level of significance. Significantly lower mean gingival scores were seen in test group as compared to control group at 90th day, the mean gingival scores of test group were 0.81 ±0.21 and control group were 1.13 ±0.23. The comparison was done using Wilcoxon paired t test, the difference reached the level of significance. Significantly lower mean gingival scores were seen in test group as compared to control group at baseline, the mean PPD of test group were 5.46 ±1.00 and control group were 5.83 ± 0.69. The comparison was done using Wilcoxon paired t test, the difference failed to reach the level of significance. At 30th day, the mean PPD of test group were 3.40 ±0.62 and control group were 4.03 ±0.80. The comparison was done using Wilcoxon paired t test, the difference reached the level of significance. Significantly lower PPD were seen in test group as compared to control group (Table 5). At 60th day, the mean PPD of test group were 2.90 ±0.60 and control group were 3.6 ±0.72. The comparison was done using Wilcoxon paired t test, the difference reached the level of significance. Significantly lower PPD were seen in test group as compared to control group at 90th day, the mean PPD of test group were 2.7 ±0.59 and control group were 3.2 ±0.86. The comparison was done using Wilcoxon paired t test, the difference reached the level of significance. Significantly lower PPD were seen in test group as compared to control group.

## Discussion:

Periodontal disease is a multi-factorial disease caused by microbial flora which is present in the subgingival plaque. *Porphyromonas gingivalis* and *Aggregatibacter actinomycetemcomitans* are the main periodontal pathogens responsible for the periodontal destruction [7]. Chronic periodontitis is more prevalent in periodontal sites exposed to these pathogens. Periodontal therapy helps in the reduction or elimination of these pathogens [8]. Nonsurgical periodontal therapy may not completely eradicate these periodontal pathogens, which leads into adjunct systemic antibiotic therapy for complete elimination of the pathogens [9] Systemic antibiotic therapy has the action of eliminating all periodontal pathogens. Some disadvantages such as inability of systemic drugs to achieve high GCF concentration, increased risk of adverse drug reactions, increased multiple antibiotic-resistant microorganisms and uncertain patient compliance [10]. These shortcomings led the invention of treatment modalities such as local drug delivery system. Pitcher *et al.* [11] observed that mouth rinses and agents used during supragingival irrigation do not predictably reach beyond 5 mm into the periodontal pocket. For antimicrobial agents to be effective the concentration of the drug should be adequate at the site and also there should be prolonged drug microbial contact. In order to overcome the drawbacks associated with systemic and conventional mode of therapy, local drug delivery systems were developed, [12] which were used in this study. A local delivery device consists of a drug reservoir and a limiting element that controls the rate of medicament release. The goal is to maintain effective concentrations of therapeutic agents at the site of action for longer period, despite drug loss from crevicular fluid clearance. Local delivery devices can be divided into two classes according to the duration of medicament release i.e., Sustained-release delivery devices and Controlled-release delivery devices [13]. Sustained-release formulations are designed to provide drug delivery for less than 24 hrs. On the other hand-controlled delivery systems have duration of drug release that exceeds 1 day. The controlled-release local delivery systems that have been used and are currently under investigation may be classified as either reservoir without a rate controlling system or with a rate controlling system as reported by Kornman *et al.* [14] Most widely used local drug delivery system reports in periodontal literature are of Tetracycline as reported by Goodson [12] and of Metronidazole by Addy *et al.* [15] Chlorhexidine by Addy *et al.* [15] and Ofloxacin by Hoffler *et al.* [16] In the present study, collagen-impregnated tetracycline fibers were used which was found to be advantageous among other drugs. Tetracyclines are preferable to other antibiotics because they are the only antibiotics that can adhere to the tooth cementum and soft tissues. They are the only antibiotics, which can achieve higher levels of gingival fluid concentrations than serum levels [17]. Tetracyclines inhibit collagenase activity, collagen degradation and bone resorption as reported by Golub *et al.* [18]. The substantivity of tetracyclines has proved to be effective against gram-positive and gram-negative anaerobic microflora associated with chronic adult periodontitis. They exert their antimicrobial effect by inhibiting protein synthesis. Tetracycline resistance may be classified into two types: nonspecific and specific.86 the former is a type of low resistance that occurs when tetracycline transport through purine channels in the outer membrane to the interior of the cell is reduced. Specific resistance can be linked to one of three mechanisms: enzymatic inactivation of drug molecules, active pump removal of tetracyclines from inside bacterial cells, or ribosome protection against tetracyclines.

Maiden *et al.* [19] reported that *in vitro* testing has shown probable periodontal pathogens including *Porphyromonas gingivalis, Fusobacterium nucleatum, Prevotella intermedia, Eikenella corrodens, Wolinella recta* and *Actinobacillus actinomycetemcomitans* are susceptible to local tetracycline concentrations achieved in periodontal pocket with a controlled release device. Therefore, tetracycline is suitable for local delivery and as an adjunct to mechanical therapy in management of periodontal disease. According to Vandekerckhove [20] treatment with tetracycline-impregnated fibers converted refractory sites to stable areas. The patients in this study were maintenance patients on 3 months recall program. Patients were monitored for clinical signs of disease evidenced by plaque, recurrent bleeding and increased probing depth. This study demonstrated that overall tetracycline fiber therapy significantly enhanced the clinical benefits obtained by SRP in chronic periodontitis patients. These results were seen for each of the key clinical parameters used to evaluate periodontitis. The benefits of SRP alone were also consistent with the results seen in other studies of periodontal disease population [21]. In the present study, A total number of 60 sites from 30 patients with periodontal pockets measuring (4-7 mm) in contra lateral quadrants were selected. Distribution of the patient among two groups showed that there was equal distribution of subjects in the age groups between 35-60. The mean age of females was 47.75± 6.29 and males were 45.3± 9.62. The comparison was done using independent t test, the difference in the age across gender failed to reach the level of significance. Intra group observations of plaque scores revealed the mean plaque scores of test group were 0.86± 0.35 and control group were 1.04± 0.34 which was statistically significant. Significantly lower plaque scores were seen in test group as compared to control group. This might be attributed to the enforced oral hygiene instructions or Hawthorne phenomena30, which might have played an important role for downward trend or reduction in the plaque score. Dang *et al.* [22] who conducted a similar study found low levels of plaque index scores when compared between SRP and SRP plus tetracycline fibers. In a similar study by Minabe *et al.* [23] observations were not in accordance with our study who found no changes in plaque scores between test group (group in which tetracycline film was placed) and control group. The gingival index score reduced significantly (p<0.001) & mean gingival index score of test group were 0.81± 0.21 and control group were 1.13± 0.23. The comparison was done using Wilcoxon paired t test. Significantly lower mean gingival scores were seen in test group as compared to control group. Similar observations were made by Minabe *et al.* [23] who found lower bleeding on probing in the group in which tetracycline film was placed. Low levels of gingival index scores were found by Dang *et al.* [22] who conducted a similar study and compared SRP and SRP plus tetracycline fibers. In another similar study by Friesen *et al.* [24] in 2002 showed that the efficacy of locally delivered tetracycline strips administered in conjunction with root planing compared to root planing alone. The author reported a significant positive PPD (p>0.033) and GI (p>0.040) reduction compared to baseline in the test group, which is in accordance with our study PPD (p>0.008) & GI (p>0.001). Sadaf *et al.* [25] in 2015 evaluated that the efficacy of tetracycline fiber (used as local drug delivery) along with scaling and root planing for the treatment of chronic periodontitis & concluded that tetracycline fiber therapy along with SRP does not improves clinical parameters PPD (p>0.05) and GI (p>0.05) significantly. This is not in accordance with our study GI (p>0.001) & PPD (p>0.001) at 30th day which might be due to short span (14 days) of study conducted by Sadaf *et al.* [25]. In our study probing pocket depth measurements showed a significant reduction in both test and the control group. There was a statistically significant difference (p<0.001) seen in the intra-group scores within each group, with greater reduction in the test group. The results were in accordance with the findings of Goodson *et al.* [10] Newman *et al.* [8] Tonetti *et al.*, [26] Dang *et al.* [22] and Minabe *et al.* [23] observed greater reduction in probing pocket depth in tetracycline plus SRP group. Within the limits of the present study, it can be concluded that locally delivered Tetracycline fiber (Periodontal Plus AB®) is safe and when used as an adjunct to scaling and root planing in chronic periodontitis showed greater improvement of clinical signs in periodontal diseases especially plaque and gingival scores and significant reduction in periodontal pocket depth as compared to scaling and root planing alone. On the basis of the clinical findings from this study, there were no adverse effects noted in the present study. This indicates that Tetracycline fiber (Periodontal Plus AB®) is a safe treatment option. Since present study is only limited to a small group, there is a need to elucidate by comparing with the larger and greater cross section of people, to evaluate the greater benefits of this local administration of antimicrobial therapy in chronic periodontitis.

## Conclusion:

Local drug administration into the periodontal pocket can enhance periodontal health while ensuring patient compliance. Local drug delivery, as opposed to systemic antimicrobials, would minimize the development of drug-resistant bacterial strains, which is now a global problem. This study exhibited that albeit careful SRP is a successful therapy technique for disposal of periodontal pockets, adjunctive utilization of locally controlled antibiotic medication tetracycline fibers has added advantage of further reducing of periodontal probing depth. Although it is still debatable whether LDD agents are cost effective in composition to systemic antimicrobial but surely it is cost effective when compared to surgical and other regeneration procedures.

## Figures and Tables

**Table 1:** Distribution of patients among two groups

**Group**	**Frequency**	**Percentage (%)**
Group 1: TEST	30	50
Group2: CONTROL	30	50

**Table 2:** comparison of mean age of patients according to gender

**Gender**	**N**	**Mean**	**Std. Deviation**	**P value**
Female	12	47.75	16.29347	0.618, Ns
Male	18	45.3333	9.62228	
Independent T test,
level of significance set at
p < 0.05 Ns: non-significant

**Table 3:** comparison of mean plaque scores among two groups at various follow up visits

**Groups**		**N**	**Mean**	**Std. Deviation**	**Mean difference**	**P value**
BASELINE	Test	30	1.1833	0.24507		
	Group					
	Control	30	1.275	0.27347	-0.09167	0.099
	Group					
30th Day	Test	30	0.5667	0.2268		
	Group					
	Control	30	0.8333	0.22102	-0.26667	.001*, Sig
	Group					
60th Day	Test	30	0.9417	0.34543		
	Group					
	Control	30	1.1417	0.30572	-0.2	.049*, Sig
	Group					
90th Day	Test	30	0.8667	0.35192		
	Group				-0.175	
	Control	30	1.0417	0.34792		0.087, Ns
	Group					
Wilcoxon paired t test;
level of significance set at p < 0.05 Ns:
non-significant Sig: significant

**Table 4:** comparison of mean gingival scores among two groups at various follow up visits

Groups		N	Mean	Std. Deviation	Mean difference	P value
BASELINE	Test		1.4417	0.23382		
	Group	30				
	Group	30				
	Group	30				
						0.052, Ns
	Control	30	1.55	0.21173	-0.1083	
	Group					
30th Day	Test	30	1.2083	0.17473		
	Group					
						0.001*, Sig
	Control	30	1.4333	0.2268	-	
	Group				0.225	
60th Day	Test	30	0.9167	0.27334		
	Group					
						0.001*, Sig
	Control	30	1.2583	0.23196	-	
	Group				0.3416	
90th Day	Test	30	0.8167	0.21709		0.001*, Sig
	Group					
						
	Control	30	1.133	0.23428	-	
	Group				0.3166	
Wilcoxon paired t test;
level of significance set at p < 0.05 Ns:
non-significant Sig: significant

**Table 5:** comparison of mean PPD among two groups at various followup visits

Groups		N	Mean	Std. Deviation	Mean difference	P value
BASELINE	Test	30	5.4667	1.00801		
	Group					
	Control	30	5.8333	0.69893	-0.3667	0.146, Ns
	Group					
30th Day	Test	30	3.4	0.62146		
	Group					
	Control	30	4.0333	0.80872	-0.6333	0.001*, Sig
	Group					
60th Day	Test	30	2.9	0.60743		
	Group					
	Control	30	3.6	0.72397	-0.7	0.001*, Sig
	Group					
90th Day	Test	30	2.7	0.59596		
	Group					
	Control	30	3.2667	0.86834	-0.5667	0.008*, Sig
	Group					
Wilcoxon paired t test;
level of significance set at p < 0.05 Ns:
non-significant Sig: significant
